# Quality assessment of mobile applications on postpartum hemorrhage management

**DOI:** 10.1590/1980-220X-REEUSP-2023-0263en

**Published:** 2024-01-08

**Authors:** Érika Maria Alves da Silva, Sheyla Costa de Oliveira, Danielle Santos Alves

**Affiliations:** 1 Universidade Federal de Pernambuco, Programa de Pós-Graduação em Enfermagem, Recife, PE, Brazil.; 2 Universidade Federal de Pernambuco, Departamento de Enfermagem, Recife, PE, Brazil.

**Keywords:** Postpartum Hemorrhage, Mobile Applications, Telemedicine, Evaluation Study, Hemorragia Posparto, Aplicaciones Móviles, Telemedicina, Estudio de Evaluación, Hemorragia Pós-Parto, Aplicativos Móveis, Telemedicina, Estudo de Avaliação

## Abstract

**Objective:**

To assess mobile application quality on the management of postpartum hemorrhage available in the digital stores of the main operating systems.

**Method:**

A descriptive evaluative study, carried out from January to February 2023 on the App Store^®^ and Google Play Store^®^. The Mobile Application Rating Scale was used to assess quality (engagement, functionality, aesthetics, information and subjective quality). Information extraction and assessment on postpartum hemorrhage was carried out using a table with information based on official documents, containing stratification, prevention, diagnosis and treatment.

**Results:**

Seven applications were included; of these, three were in English, six had an Android operating system. The quality mean was 3.88. The highest means were for functionality, reaching 5.0 (n = 6), and the lowest were for engagement, less than 3.0 (n = 4). The majority of applications presented less than 50% of the information on postpartum hemorrhage management.

**Conclusion:**

The applications assessed achieved an acceptable quality mean and, according to health organizations’ current protocols, did not contain the necessary information for complete postpartum hemorrhage management.

## RESUMO

**Objetivo:**

Avaliar a qualidade dos aplicativos móveis sobre o manejo da hemorragia pós-parto disponíveis nas lojas digitais dos principais sistemas operacionais.

**Método:**

Estudo descritivo de avaliação, realizado de janeiro a fevereiro de 2023 nas lojas digitais *App Store*® e *Google Play Store*®. Foi utilizada a *Mobile Application Rating Scale* para avaliação da qualidade (engajamento, funcionalidade, estética, informação e qualidade subjetiva). A extração e a avaliação das informações sobre hemorragia pós-parto foram realizadas a partir de um quadro com informações baseadas em documentos oficiais, contendo a classificação, prevenção, diagnóstico e tratamento.

**Resultados:**

Sete aplicativos foram incluídos; desses, três estavam em inglês, seis tinham sistema operacional *Android*. A média de qualidade foi de 3,88. As maiores médias foram da funcionalidade, alcançando 5,0 (n = 6), e as menores foram de engajamento, menos que 3,0 (n = 4). A maioria dos aplicativos apresentou menos de 50% das informações sobre o manejo de hemorragia pós-parto.

**Conclusão:**

Os aplicativos avaliados alcançaram média de qualidade aceitável e, conforme os protocolos vigentes das organizações de saúde, não continham as informações necessárias para o manejo completo da hemorragia pós-parto.

## RESUMEN

**Objetivo:**

Evaluar la calidad de las aplicaciones móviles sobre el manejo de la hemorragia posparto disponibles en las tiendas digitales de los principales sistemas operativos.

**Método:**

Estudio de evaluación descriptivo, realizado de enero a febrero de 2023 en las tiendas digitales App Store^®^ y Google Play Store^®^. Se utilizó la Escala de Calificación de Aplicaciones Móviles para evaluar la calidad (compromiso, funcionalidad, estética, información y calidad subjetiva). La extracción y evaluación de la información sobre la hemorragia posparto se realizó mediante una tabla con información basada en documentos oficiales, que contiene clasificación, prevención, diagnóstico y tratamiento.

**Resultados:**

Se incluyeron siete aplicaciones; de ellos, tres estaban en inglés, seis tenían sistema operativo Android. El promedio de calidad fue 3,88. Los promedios más altos fueron para la funcionalidad, alcanzando 5,0 (n = 6), y los más bajos fueron para el compromiso, menos de 3,0 (n = 4). La mayoría de las solicitudes presentaron menos del 50% de la información sobre el manejo de la hemorragia posparto.

**Conclusión:**

Las aplicaciones evaluadas alcanzaron un promedio de calidad aceptable y, según los protocolos vigentes de las organizaciones de salud, no contenían la información necesaria para el manejo completo de la hemorragia posparto.

## INTRODUCTION

Access to technology through mobile devices is increasing every year. Report shows that Brazil reached 258.3 million cell phones in 2022^(1)^. Consequently, access and downloads of applications (apps) increased considerably, placing Brazil as the fourth country that downloaded the most apps, according to the State of Mobile 2022 report^(2)^.

The popularization of smartphones is considered a technological revolution of great impact. Using mobile technologies, such as apps, for healthcare and information access purposes, is a promising form of intervention, considering cost-effectiveness, scalability and high reach. Mobile computing can be applied in various aspects within the healthcare area, such as remote monitoring and training professionals^(3)^.

Using these technologies for health information and care promotion is defined as mHealth^(4)^, and contributes to reducing difficulties related to geographic barriers in healthcare and the provision of easily understood knowledge. Its potential includes support for clinical diagnosis, decision-making, behavior change, autonomous digital therapy and disease-related education^(5)^.

The development of good quality health applications that work to change practices is one of the recommendations of the Digital Health Strategy (DHS28) for Brazil. Among the objectives of DHS28 are innovation initiatives, service models, knowledge extraction mechanisms and digital health apps originating from user needs^(6)^.

Increasingly, studies are being conducted on apps for important health topics such as cancer, sexually transmitted infections, and pregnancy^(7–9)^. Using mHealth in maternal health is a current reality and covers different areas of the pregnancy-puerperal cycle, offering information about pregnancy^(10)^ and aspects of childbirth and the postpartum period^(11)^.

Obstetric complications are also addressed in the apps, such as postpartum hemorrhage (PPH), as it is one of the main causes of maternal morbidity and mortality in the world^(12)^. Authors assessed the effect of a training application on nurses’ and midwives’ knowledge and skills for PPH management and neonatal resuscitation, and found that knowledge and skill scores increased significantly after its use^(13)^.

PPH is a relevant topic in the context of public health. Almost a quarter of maternal deaths worldwide are associated with this complication, being the first cause in low-income countries^(14)^. The United Nations (UN) emphasizes improving access to technologies and recommends that countries should integrate digital health and mHealth into their national health information systems and health infrastructure^(15)^.

It is important that studies be developed to assess health application quality, as their content may influence users’ decision-making. The rapid increase in the number of smartphone applications makes this assessment increasingly necessary, as it is difficult to identify high-quality applications and the security of their information sources^(16)^.

Applications aimed at pregnancy, for the most part, are of low quality^(17)^ and present different content, however, in a fragmented way, and few present the sources^(18)^. When assessing applications, criteria such as appearance, structure, navigation, reliability and content are generally used^(19)^. However, assessing mHealth application quality requires specific criteria inherent to their development and content^(16)^.

Therefore, in order to assess mHealth applications on PPH, the following research question was raised: what is the quality of the mobile applications on the management of PPH available in the digital stores of the main operating systems? This study aimed to assess mobile application quality on PPH management available in the digital stores of the main operating systems.

## METHOD

### Study Design

This is a descriptive and evaluative study, conducted in six stages: 1) Definition of assessment objectives; 2) Establishment of application inclusion and exclusion criteria; 3) Selection of information to be extracted; 4) Search for applications and analysis of the results obtained; 5) Presentation of assessment results; 6) Discussion of results^(20)^. This research is premised on carrying out a systematic assessment guided by a validated instrument and following a research protocol for a structured search.

### Data Collection Site

The search for apps was carried out from January to February 2023, in App Store^®^ (iOS) and Google Play Store^®^ (Android), using two mobile devices that support the aforementioned operating systems: Xiaomi Redmi Note 10 version 13.0.11 (Android) and an iPhone 7 version 15.7.2 (iOS) device. The following search terms were used, individually, in English and Portuguese*: hemorragia puerperal, risk stratification, parto seguro, hemorragia* and *pós-parto* (postpartum hemorrage, risk index, safe delivery, hemorrhage e postpartum).

### Selection Criteria

Free apps were included, aimed at healthcare professionals, compatible with Android and/or iOS operating systems and that mentioned in their title or description obstetric emergencies and/or PPH, and that covered in their content information about PPH management. Paid apps, those that required institutional login and those that were temporarily disabled were excluded. Two searches were carried out in each digital store, by two researchers, until ratifying the inclusion of applications.

### Data Collection

Access to app information and content occurred by checking the data available in the digital stores themselves and by downloading them directly to mobile devices (cell phones). From there, the application was accessed, and all its content was fully explored by the authors, examining the information about PPH, the gaps and the way the content was presented.

**Figure 1:**
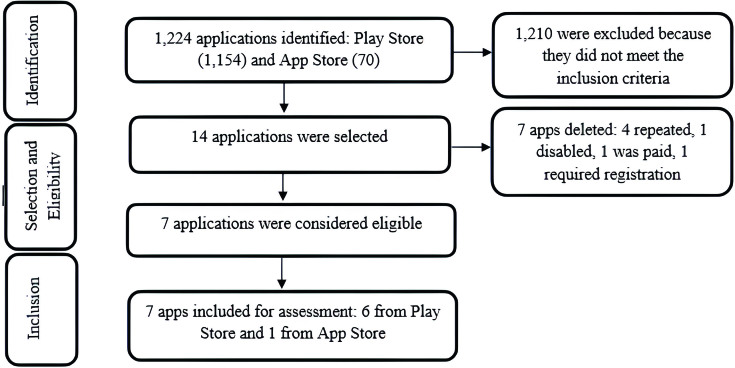
Application screening and selection flowchart based and adapted from the Transparent Reporting of Systematic Reviews and Meta-analyses (PRISMA)^(21)^ regarding the search and selection process. Recife, PE, Brazil, 2023.

Figure 1 reflects the screening and selection process for applications. During data collection, 1,224 apps were identified by including search terms. Of these, 1,210 were excluded because they did not meet the inclusion criteria. Thus, 14 applications were selected to assess the eligibility criteria. Of these, seven were excluded, as four were repeated, one was temporarily disabled, one was paid and one needed registration. Seven applications were included in the sample: six extracted from Google Play Store^®^ and one from App Store^®^.

To assess the apps, a validated instrument was used, developed specifically to assess mHealth apps: the Mobile Application Rating Scale (MARS)^(16)^. It consists of 19 objective items and 4 subjective items, and assesses engagement, functionality, aesthetics, information and subjective quality. Each area has a score ranging from one to five, obtaining a mean and, in the end, an overall quality mean, with the subjective area being assessed separately (1 = Inadequate, 2 = Poor, 3 = Acceptable, 4 = Good and 5= Excellent).

To assess information about PPH in the apps, a table was developed consisting of 20 pieces of information related to PPH management, divided into four categories: 1) Definitions/stratifications; 2) Prevention; 3) Diagnosis; 4) Treatment. Each one is made up of five pieces of information, which are equivalent to 100% of the expected quantity for the category. Therefore, each item is equivalent to 20%, and, at the end, the percentage of information present is calculated. Data regarding PPH management were extracted from official documents from national and international healthcare organizations^(12,22,23)^.

### Data Analysis and Treatment

Data analysis was carried out descriptively and quantitatively, after assessing the app quality and reading and extracting the main information about PPH. From the analysis, the results were discussed according to the MARS quality assessment criteria and based on scientific literature on the subject.

### Ethical Aspects

For the present study, assessment by an ethics committee was not necessary, given its defining characteristics, in accordance with current regulations. Furthermore, the study did not involve the participation of any human volunteers as invited research participants for any of its stages.

## RESULTS

The most prevalent language was English (n = 3), and an app was developed in Portuguese. An app was customizable in terms of language, with 30 versions available depending on the country. Six apps had the Android operating system. The majority had their last update carried out in 2022 (n = 4), and there were no user reviews in digital stores (n = 5). Two apps had more than 10,000 downloads and one had more than 100,000. The largest size of the apps was 54.83 MB while the smallest was 11.1 MB (Chart 1).

**Chart 1:** Description of selected applications regarding their development characteristics, language, operating system, update, assessment, download and size – Recife, PE, Brazil, 2023.

Name	Developer	Language	Operational system	Update month/year	Review score*/number of reviews	Number of downloads	Size
Maternal & Newborn Care Plans	AFRA Dev	English	*Android*	December 2022	0/0	>10,000	11.1 MB
Postpartum Hemorrhage	Prof. Pinki Shrivastava	English	*Android*	April 2022	0/0	>100	7.88 MB
*Risco Hemorrágico Obstétrico*	Juliano Gaspar (*Faculdade de Medicina de Minas Gerais* - UFMG)	Portuguese	*Android*	November 2018	5/18	>50	21.93 MB
*El embarazo y el parto seguros*	Hesperian health guides	Spanish	*Android*	March 2017	3.4/110	>10,000	14.15 MB
*GPCs Ginecología y Obstetricia*	Jorge Madrigal Veja	Spanish	*Android*	January 2021	0/0	>50,000	54.83 MB
Safe delivery	Maternity foundation	Customizable (30 versions)	*Android*	October 2022	0/0	>100,000	11.85 MB
ACOG DII SMI	Fountainhead Mobile Solutions	English	iOS	February 2022	0/0	**	41.1 MB

Source: research data.

*The assessment score refers to the one available in the store where it was extracted and ranges from 0 to 5 stars. **It was not possible to access the number of downloads, as it was not available on the platform (zero?).

In application assessment by MARS, the highest means were obtained in functionality (4.88) and the lowest in engagement, with two reaching a mean above 3.0 and one above 4.0. No application achieved an overall quality mean of 5.0, and three achieved means greater than 4.0 (Chart 2). In the subjective quality assessment, an application received a mean of 5.0 points. The rating given by users to apps in digital stores was similar to the MARS quality mean.

**Chart 2:** Application assessment according to engagement, functionality, aesthetics, information and subjective quality – Recife, PE, Brazil, 2023.

APPLICATION NAME	MOBILE APPLICATION RATING SCALE (MARS) SCORE					
	Engagement mean	Functionality mean	Aesthetics mean	Information mean	Overall quality mean	Subjective quality mean
Maternal & Newborn Care Plans	2.2	5.0	3.7	3.4	**3.57**	2.2
Postpartum Hemorrhage	2.2	5.0	3.3	3.0	**3.37**	1.5
*Risco Hemorrágico Obstétrico*	3.2	5.0	3.7	4.4	**4.07**	4.2
*El embarazo y el parto seguros*	2.8	5.0	3.0	3.7	**3.62**	2.5
*GPCs Ginecología y Obstetricia*	2.4	4.5	4.0	4.5	**3.85**	2.2
Safe delivery	4.6	5.0	5.0	4.1	**4.67**	5.0
ACOG DII SMI	3.4	4.7	3.7	4.3	**4.02**	4.0
**Rating mean**	**2.97**	**4.88**	**3.85**	**3.91**	**3.88**	**3.37**

Note: the mean subjective quality was assessed separately so as not to interfere application quality mean, due to its subjective nature.

The contents on PPH management^(12,22,23)^ were assessed by categories, as follows:

1) Definition/stratifications: PPH (vaginal), PPH (cesarean section), massive PPH, primary PPH, secondary PPH;
2) Prevention: risk factors, antepartum risk stratification, intrapartum risk stratification, preventive care measures (timely umbilical cord clamping, controlled umbilical cord traction, Brandt-Andrews maneuver, uterine massage, mother-child skin-to-skin contact, rational use of oxytocin during labor, not performing the Kristeller Maneuver), preventive medication measures (oxytocin 10 IU/intramuscular after birth);
3) Diagnosis: visual estimation, weighing pads/pads, collection devices, clinical parameters, shock index;
4) Treatment: medication (oxytocin, tranexamic acid, methylergometrine, misoprostol), non-surgical (uterine massage, intrauterine tamponade balloon, non-pneumatic anti-shock suit), surgical (compressive sutures, vascular sutures, hysterectomy, damage control), treatments for other causes (trauma, thrombin and tissue), care procedures (elevating lower limbs, supply of O^2^, indwelling urinary catheter, monitoring).

Chart 3 displays the percentage of information on PPH management present in the applications included in the sample. Of the apps assessed, 71.4% (n = 5) presented less than 50% of the information and two apps presented less than 30%.

**Chart 3:** Percentage of information on postpartum hemorrhage management and information present and absent in the applications included in the sample – Recife, PE, Brazil, 2023.

INFORMATION ABOUT PPH MANAGEMENT	APPLICATION NAME						
	Maternal & Newborn Care Plans	Postpartum Hemorrhage	*Risco Hemorrágico Obstétrico*	*El embarazo y el parto seguros*	*GPCs Ginecología y Obstetricia*	Safe Delivery	ACOG DII SMI
	Percentual de informações presentes por categoria						
**DEFINITION/STRATIFICATIONS**	**60%**	**60%**	**0%**	**20%**	**20%**	**0%**	**0%**
PPH (vaginal)	P	P	–	P	P	–	–
PPH (caesarian)	–	–	–	–	–	–	–
Massive PPH	–	–	–	–	–	–	–
Primary PPH	P	P	–	–	–	–	–
Secondary PPH	P	P	–	–	–	–	–
**PREVENTION**	**20%**	**20%**	**100%**	**0%**	**60%**	**40%**	**100%**
Risk factors	–	–	P	–	P	–	P
Antepartum RS	–	–	P	–	–	–	P
Intrapartum RS	–	–	P	–	–	–	P
PM care	P	–	P	–	P	P	P
PM medications	–	P	P	–	P	P	P
**DIAGNOSIS**	**40%**	**20%**	**0%**	**40%**	**60%**	**0%**	**60%**
Visual estimation	–	–	–	P	P	–	P
Weighing pads/pads	P	–	–	–	–	–	P
Collecting devices	–	–	–	–	–	–	P
Clinical parameters	P	P	–	P	P	–	–
Shock Index	–	–	–	–	P	–	–
**TREATMENT**	**60%**	**40%**	**0%**	**40%**	**60%**	**80%**	**80%**
Medicinal	P	P	–	P	P	P	P
Non-surgical	–	–	–	–	P	P	P
Surgical	–	–	–	–	P	–	P
Trauma, thrombin and tissue treatments	P	–	–	–	–	P	–
Assistance behaviors	P	P	–	P	–	P	P
**% TOTAL INFORMATION**	**45%**	**35%**	**25%**	**25%**	**50%**	**30%**	**60%**

**Source**: research data. **Caption:** PPH = postpartum hemorrhage; P = present; – = absent; RS = risk stratification; PM = preventive measures.

## DISCUSSION

According to MARS, applications considered excellent (mean 5) must contain: engagement (fun, interesting, customizable, interactive, sending alerts, messages, reminders, feedback); good functioning (easy learning and navigation, flow logic and gestural design); pleasing aesthetics (graphic design, visual appeal, color scheme and stylistic consistency); quality information (adequate text, good extensiveness, feedback, references, credibility); and a good subjective quality assessment, which involves interest in using and recommending the app. The absence or deficiency of such aspects leads to a reduction in its score and overall quality rating^(16)^.

The mean quality of the applications assessed in this study was 3.88, rated as acceptable quality. Apps for pregnancy and postpartum have an overall quality mean of 3.06 according to the same instrument, lower when compared to health apps in general (3.74). Among the items, functionality is the area with the best assessment in the applications and stands out with higher means, while engagement, information and aesthetics have lower means^(24,25)^.

“Safe Delivery”, one of the apps assessed, received the highest mean in quality assessment (4.67) and proved to be a dynamic, interesting and interactive application, with explanatory videos, knowledge tests, user input and customizations regarding the language and user profile, with content suited to the target audience (engagement). Furthermore, it did not exhibit any malfunction, connectivity, with good layout and graphics (functionality and aesthetics).

“*Risco Hemorrágico Obstétrico*”, with a quality rating of 4.07, it was developed by researchers from the *Universidade Federal de Minas Gerais*, with the aim of carrying out risk stratification for PPH of pregnant women in the antepartum and intrapartum periods. It has a clean and easy-to-use design, but with few interaction features and does not allow customization. In the app, risk factors are classified as medium and high risk, with no low risk stratification, as recommended by the Pan American Health Organization and the World Health Organization^(22)^.

“ACOG DII SMI” has broad content, with small functional flaws (some links/buttons do not lead to the proposed content and show an error signal) and an overall mean rating of 4.02. It is not very easy to use, requiring many clicks to reach the main result as well as little interactivity. It was developed with the aim of providing standardized approaches to maternal and child health. Most of its content is presented in text and slide format, with some checklists and few images. These facts justify the lower means in aesthetics and engagement.

User interface visual design is one of the important points in app development. Research carried out to analyze the user interface design of 88,861 apps in the App Inventor gallery showed that the majority did not comply with design guidelines and did not have good aesthetics^(26)^. Overall app development requires attention from the developer community. Users of mHealth apps in maternal health report a greater likelihood of using apps that are aesthetically pleasing and have minimal technological barriers^(27)^.

Regarding information accuracy in the app description, the presence of goals/objectives, the quality and scope of the information covered, presence of visual information, credibility and evidence base used, the apps assessed in this study obtained quality means between 3.62 and 4.67, similar to those of other studies^(25)^. Efforts are recommended in developing content for apps focused on improving the quality of health applications, in order to bring about changes in user practices^(17)^.

Ratings carried out on apps about pregnancy showed that the majority had flaws in the quality of their information, without scientific evidence or citation of their content sources. Furthermore, it was observed that the focus was maintained on functionality. All lacked transparency regarding affiliations, i.e. they did not inform whether the app development was associated with any public or private institution, or the author’s own development^(28)^.

“Maternal & Newborn Care Plans”, “Postpartum Hemorrhage” and “*GPCs Ginecología y Obstetricia*” were the apps that obtained the lowest mean in terms of engagement, with scores between 2.2 and 2.4 being attributed. The low mean engagement attributed to Maternal & Newborn Care Plans may be a reflection of the presentation format of its content, at the time, presented in text, with few interactive elements, without images and without personalization.

Achieving good user engagement with the technology offered is essential for interventions and health behavior change to be effective. This problem can be solved by incorporating more customizable features, with more attractiveness and options that improve application interactivity, making it easier to use for longer periods of time^(29)^.

“Postpartum Hemorrhage”, developed for cases of PPH management in the primary center and safe referral to a tertiary center, has a simple design, with almost no interaction with users, content available in text, flowchart and some illustrative images of the topics covered, having been rated as “poor” in engagement (mean 2.2). However, its mean functionality was excellent (5.0).

With regard to evidence base assessment (if the application was tested/verified by evidence), guided by MARS^(16)^, only studies that involved assessing “Safe Delivery” were found, with these positive results in relation to its effectiveness in improving professionals’ knowledge in PPH management and neonatal resuscitation^(13)^.

Regarding the topics about PPH covered in the apps, most focused their content on bleeding disorder treatment. Guidance flows, medications to be offered and conduct in more serious cases were presented, such as using intrauterine tamponade balloon, compressive sutures and hysterectomy. Information regarding prevention, risk factor screening and risk stratification for PPH was less covered.

The list of the main risk factors for PPH was present in the applications “*GPCs Ginecología y Obstetricia*”, “*Risco Hemorrágico Obstétrico*” and “ACOG DII SMI”. However, risk stratification was presented in only two of them. National and international health organizations recommend that risk factors for PPH be investigated in all pregnant women since prenatal care as well as carrying out risk stratification so that appropriate and preventive measures are taken for each case^(12,22,23)^.

Some applications may have presented a low percentage of information on PPH management due to the purpose for which it was intended. As an instance, “*Risco Hemorrágico Obstétrico*” is cited, which was developed solely for risk stratification of PPH, which may have led the developers to believe that it was not necessary to include information on definitions and treatment.

Only one app assessed addressed the Shock Index (SI), with clear and objective information about its values and the interpretation of its results. SI is an early predictive value of hemodynamic instability in PPH^(22)^, being a consistent predictor in comparison with conventional means in PPH^(30)^.

It is emphasized that the most prevalent information in the apps (those related to treatment) is of great relevance, as correct and effective treatment minimizes the chances of maternal morbidity and mortality, favoring a good prognosis. However, missing and/or incomplete information (definitions, prevention and diagnosis) demonstrate a dassessment of measures that can prevent or predict cases of PPH, guiding the conduct to be followed.

From the above, it is clear that ratings of technological tools developed for maternal health regarding their effectiveness and general quality need to be implemented to guarantee the security of the information offered. They appear to be a potentially effective strategy for changing behavior, needing to encompass aspects such as engagement and aesthetics, good interactivity and images, with pleasant aesthetics and minimal technological barriers.

The main gap identified in this study was that none of the applications addressed the essential information for complete PPH management in a unified way, requiring users to download more than one app. The main limitation of this study was restricted access to some applications that required institutional login or were paid, not allowing for content and quality assessment.

## CONCLUSION

In quality assessment, the applications achieved acceptable quality. Engagement and aesthetics had the lowest mean ratings. Regarding the extent of information, the majority presented a low percentage of information on PPH in accordance with what is recommended by national and international healthcare organizations. Only one of the seven applications assessed was tested through a scientific study.

It is recommended that the applications developed present a quality assessment and prioritize information that meets the target population’s knowledge demand. Good quality apps, with comprehensive content based on good practices and scientific evidence, can have a positive impact on qualified care in obstetric care and professional decision-making, for concrete and effective ongoing education.
